# Kinetic Modeling of Brain [^18-^F]FDG Positron Emission Tomography Time Activity Curves with Input Function Recovery (IR) Method

**DOI:** 10.3390/metabo14020114

**Published:** 2024-02-08

**Authors:** Marco Bucci, Eleni Rebelos, Vesa Oikonen, Juha Rinne, Lauri Nummenmaa, Patricia Iozzo, Pirjo Nuutila

**Affiliations:** 1 Turku PET Centre, Turku University Hospital, 20521 Turku, Finland; 2 Turku PET Centre, University of Turku, 20521 Turku, Finland; 3 Turku PET Centre, Åbo Akademi University, 20521 Turku, Finland; 4 Theme Inflammation and Aging, Karolinska University Hospital, SE-141 86 Stockholm, Sweden; 5 Division of Clinical Geriatrics, Department of Neurobiology, Care Sciences and Society, Center for Alzheimer Research, Karolinska University, SE-141 84 Stockholm, Sweden; 6 Department of Psychology, University of Turku, 20520 Turku, Finland; 7 Institute of Clinical Physiology (IFC), National Research Council (CNR), 56124 Pisa, Italy; 8 Department of Endocrinology, Turku University Hospital, 20521 Turku, Finland

**Keywords:** input function, Feng input, Bayesian estimation, positron emission tomography, kinetic modeling, hyper-insulinemic euglycemic clamp, fluorodeoxyglucose, brain

## Abstract

Accurate positron emission tomography (PET) data quantification relies on high-quality input plasma curves, but venous blood sampling may yield poor-quality data, jeopardizing modeling outcomes. In this study, we aimed to recover sub-optimal input functions by using information from the tail (5th–100th min) of curves obtained through the frequent sampling protocol and an input recovery (IR) model trained with reference curves of optimal shape. Initially, we included 170 plasma input curves from eight published studies with clamp [^18^F]-fluorodeoxyglucose PET exams. Model validation involved 78 brain PET studies for which compartmental model (CM) analysis was feasible (reference (ref) + training sets). Recovered curves were compared with original curves using area under curve (AUC), max peak standardized uptake value (maxSUV). CM parameters (ref + training sets) and fractional uptake rate (FUR) (all sets) were computed. Original and recovered curves from the ref set had comparable AUC (d = 0.02, not significant (NS)), maxSUV (d = 0.05, NS) and comparable brain CM results (NS). Recovered curves from the training set were different from the original according to maxSUV (d = 3) and biologically plausible according to the max theoretical K1 (53//56). Brain CM results were different in the training set (*p* < 0.05 for all CM parameters and brain regions) but not in the ref set. FUR showed reductions similarly in the recovered curves of the training and test sets compared to the original curves (*p* < 0.05 for all regions for both sets). The IR method successfully recovered the plasma inputs of poor quality, rescuing cases otherwise excluded from the kinetic modeling results. The validation approach proved useful and can be applied to different tracers and metabolic conditions.

## 1. Introduction

Positron emission tomography (PET) with the glucose analogue tracer ^18^F-fluorodeoxyglucose ([^18^F]FDG) is the current gold standard technique for non-invasive in vivo measurement of brain glucose metabolic rate [1,2]. There are several ways of analyzing [^18^F]FDG data, and these vary depending on the research question. In metabolic research imaging, our group [3,4,5,6,7,8] and others [9,10,11,12,13] have typically used quantified measurements of the PET data to estimate the brain glucose metabolic rate via graphical approaches (such as the Gjedde–Patlak plot [14,15] and its approximate fractional uptake rate (FUR) [16,17,18]) or via compartmental kinetic modeling [2,19]. The quantification of the PET data requires the measurements of the tracer quantity in the plasma from the time of injection of the tracer until the end of the study, and thus, either arterial or arterialized (i.e., heated venous blood) samples are drawn throughout the study. Alternatively, the input function must be derived from the PET images, for instance, from the left ventricle of the heart [20,21,22,23,24] or from the carotids, often aided by magnetic resonance images [25,26,27,28,29,30] and some with the employment of simultaneous estimation (SIME) approach [31,32,33,34,35] (for reviews, please refer to [34,36]).

Unfortunately, during a PET study, sampling from an arterialized vein (which is very often preferred over an arterial cannulation to avoid extra discomfort for the volunteer) can fail due to several reasons. The volunteer may experience discomfort, mild claustrophobia and stress. Fear can induce vasoconstriction, and even in well-warmed veins, sampling can prove to be very challenging. On the other hand, the kinetics of the tracer in the blood are fast, as, immediately after the tracer injection, the tracer distributes into the whole vascular volume, and it is actively taken up from the tissues. It follows that inappropriate sampling, even for just few minutes following the tracer injection, can jeopardize the quality of the whole PET study and of its quantification, especially with compartmental modeling (CM) analysis—because it is so sensitive to the initial peak shape. A potential solution to this problem is to scan the chest area first, since the placement of a small region of interest (ROI) in the left ventricle of the heart, can adequately give the input function, and the frequent sampling, necessary to properly resolve the peak of the input function, can be avoided [20,21,22,23]. However, the PET study must often start directly from the targeted tissue to obtain the time activity curve of the tissue from the injection, necessary for quantitative methods (such as, compartmental modeling). Thus, the reliance on the plasma input curves is the only alternative, despite the probable problems of sampling of the peak at the very beginning of the curve.

The input curve consists of a peak (with steep ascending and descending phases between 0 and approximately 2 min after the tracer administration) and a tail portion. It has been pointed out how difficult it is to find a single analytical formulation able to produce a good model for fitting both the peak and tail of the input function [36,37]. Both groups of Guo et al. [37] and Sanabria-Bohorquez et al. [31] used a hybrid approach employing the use of image-derived input data for the peak and tail, respectively, and the other part was estimated from blood samples.

The aim of this study was to propose a new solution in “recovering” the peaks of input function in cases where blood sampling during the beginning of an [^18^F]FDG-PET study has failed. To this end, plasma input curves considered to be of poor quality were “recovered” utilizing the tail portion to recover the peak. 

In the proposed method, reference curves are used to initially validate the recovery function. Many studies have already taken advantage of population-based input functions which are appropriately scaled standard input functions based on the population pool of representative curves and often used in combination with the blood samples of the tail [36].

The process of input recovery aims to correct (“recover”) the peak of poor-quality curves. The tails of the poor-quality curves (from 5th to 100th min after tracer injection) are fitted with an algorithm that employs a previously published equation to describe [^18^F]FDG input functions by Feng et al. [38], included in a pre-validated input estimation model powered by a Bayesian penalized-likelihood term and an additional set of equations trained with reference data. The Bayesian penalized-likelihood used in our fitting algorithm has been implemented in the past and gradually integrated in the newest reconstruction algorithms for PET/CT scanners [39,40,41]. Based on such improved reconstructions, input functions have been derived from images [42]. But only the new PET/CT scanners provide a resolution and new algorithms that allow the extraction of input from images. Old scanners must be coupled with blood sampling to a certain extent. 

Finally, to ensure the biological plausibility of the newly obtained recovered curves, we calculate the theoretical maximal K1 parameter from compartmental modeling. This was obtained based on the cerebral blood flow measured in the reference set images of [^18^H]H_2_O (gold standard technique) [43].

Our method is hereby summarized. First, we validate it on a reference set of input curves. Second, we select poor-quality curves from the training set via criteria validated against the reference set and recover the inputs. Thirdly, based on the reference set (good-quality, n = 13) and “recovered” input functions (poor-quality, n = 56), we apply compartmental modeling to the brain tissue curves for the subjects for which dynamic data allowed the kinetic modeling, while FUR can be computed for the subjects whose brains were scanned about one hour after the injection (late scans). We sought to show that the method presented using almost the entirety of the curves (from 5th to 100th min), produces recovered curves comparable to the reference ones (with an optimal shape according to the predefined constraints and almost solely from arterial sampling) and yields comparable and biologically plausible compartmental modeling results. 

## 2. Materials and Methods

### 2.1. Subjects

We pooled and re-analyzed all applicable [^18^F]FDG brain studies carried out under euglycemic hyperinsulinemic clamp (n = 170) at the Turku PET Centre performed between 2004 and 2016, which were also included in a recent publication [44]. The anthropometric and metabolic characteristics of all study participants are listed in Table 1 (and Table S1), Tables S2 and S3 (grouped by dataset, input quality (from criteria validated in step 5 of the validation process) and input source, respectively). Each study protocol included in this study was approved by the Ethics Committee of the Hospital District of Southwest Finland and conducted in accordance with the Declaration of Helsinki, and each participant gave written consent prior to inclusion in each study. 

### 2.2. PET Study during Clamp

The euglycemic hyperinsulinemic clamp was performed as previously described [45]. Subjects, which had fasted for 10–12 h, were admitted to our facilities, and cannulated for easy access for blood sampling and liquid infusions. A primed-continuous infusion of insulin (Actrapid; Novo Nordisk, Copenhagen, Denmark) was given (40 mU^.^m^−2.^min^−1^) and a variable rate of a 20% glucose solution was infused to maintain euglycemia (5 mmol/L). Plasma glucose levels were measured every 5–10 min throughout the clamp. At approximately 100 ± 10 min into the clamp, [^18^F]FDG (187 ± 30 MBq, mean ± SD) was injected intravenously over 15 s and the brain radioactivity started either immediately after (n = 78, “early” studies), or approximately 1 h after [^18^F]FDG injection (n = 92, “late” studies). The PET study protocols are described in more detail in previous reports [3,4,5,6,7,8,46,47]. The reason for which some studies performed the brain scans early or late is due to the design of usually multi-organ PET studies, which might a have different focus on which is the main organ of interest to study at the beginning of the PET acquisitions with better quantification options. Among the standard procedures of PET acquisition, both the PET image and sample input function have been corrected for radioactive decay.

### 2.3. Study Design

Our “early” studies (n = 78) were originally selected as the population to train and validate the model since compartmental model analysis requires the whole tissue–time–activity curves from time 0 from the injection, and these studies provide them.

The “late” studies (n = 92) were used as a test dataset and only the fractional uptake rate analysis was possible. 

**Figure 1:**
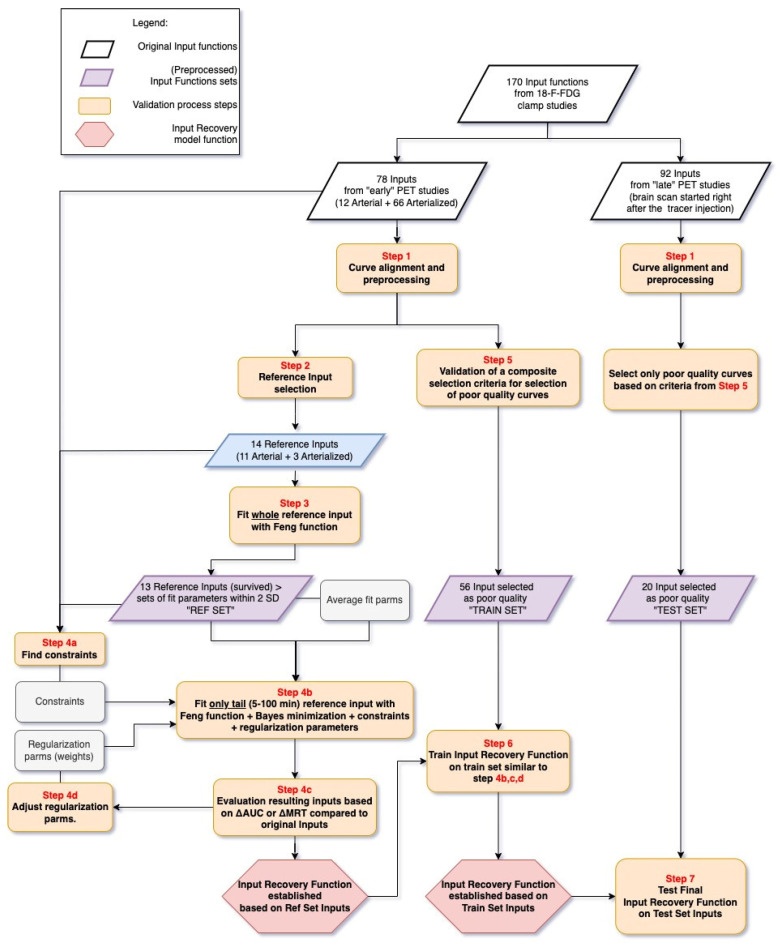
Validation process of the input recovery (IR) method step-by-step.

Input functions were obtained from three types of sources: arterial samples (whole curve), arterialized samples (whole curve) and peak from image (aortic arch or left ventricle) + tail from arterialized samples (Table S3). The validation process of the input recovery model is illustrated in Figure 1. All the steps are described in the following paragraph. In brief, selected reference input curves were used to train an input recovery model that is applied to poor-quality inputs. The quality of the input is mainly defined by the height and shape of the activity peak. The model, using the averaged fitting parameters of the input function equation and a few constraints, recovers the input peak using just the tail (5 min–100 min). The model trained on the reference inputs was additionally tuned in its weightings based on the poor-quality curves (selected based on a visual assessment validation criteria) to ensure that all fits were acceptable. The model has been subsequently tested on the late poor-quality input functions.

### 2.4. Input Recovery (IR) model—Validation Steps

*(1) Peak alignment and saving of the delay to generate peak–aligned curves (preprocessed).*

*(1a) Excluding very low (or negative) activity values at the beginning of the curve.* In the whole dataset of 78 input curves, we first obtained the median of the 1st non-zero activity data points, which was 0.074 kBq/mL. All curves that had 1st non-zero activity data points <0.074 kBq/mL were rounded to 0. Then, for each curve, the time of the maximum peak was determined. In cases where two peaks were nearly equal (less than 10% of variation between the two peaks), the mid-time between them was used. 

*(1b) Identification of the start of the ascending phase of the input curve.* When only two points were present between the 1st non-zero activity data points (A) and the peak, then the “0” point was extrapolated by applying a linear regression. On the contrary, if >2 points were between the 1st non-zero activity data points (A) and the peak, then a polynomial fit was applied.

*(1c) Identification of population delay time.* The max peak time of all input curves were plotted, and the upper adjacent value (UAV) was identified. UAV is the largest observation that is less than or equal to the upper inner fence (UIF), which is the third quartile plus 1.5*Interquartile range [48]. All peak times that are less than the UAV time (UAV*t*) were delayed to UAV*t*, obtaining the peak-aligned (PA) inputs and the delay time (dt) were stored. These delay times were used again later (steps 6 and 7) to bring back the input curve to the original times. 

*(2) Selection of the reference peak–aligned Inputs.*

The max activity at the peak of the PA inputs was divided by the activity at time point UAV*t* + 2.5 min (RT), and the value between the 3rd and 4th quartiles was identified and used as ratio threshold (RT_1_). Then, whole inputs that had a RT ≥ RT_1_ and time of max activity ≤ UAV*t* + 1 and max peak ≥ 50 kBq/mL were selected as reference inputs (n = 14).

*(3a) Fit with Feng function of reference PA inputs (whole curve).*

The reference PA inputs were fitted according to a previously published function from Feng et al. [38], for t ≥ τ.

(1) $$\left[A_{1}\left(t-\tau \right)-A_{2}-A_{3}\right]*e^{\lambda_{1}*\left(t-\tau \right)}+\left[A_{2}\right]*e^{\lambda_{2}*\left(t-\tau \right)}+\left[A_{3}\right]*e^{\lambda_{3}*\left(t-\tau \right)}$$

The Feng input function fits the shape of the [^18^F]FDG input curve using three exponentials and seven parameters (A_1–3_, λ_1–3_ and τ) (code of the equation is reported in Supplemental Material and Methods) via Matlab, using an optimization procedure (lsqcurvefit and Levenberg–Marquardt algorithm) that minimized the difference between the original and fitted activity time points. The fits were visualized as superimposed to the original curves and the area under the curve (AUC) and mean residence time (MRT) were computed to evaluate the differences between the curves [49]. MRT is a parameter imported from the pharmacology studies that is useful for time–activity comparison, and calculated via the area under the first moment curve (AUMC), as described below:

MRT = AUMC/AUC (2)

(3) $$AUMC=\int_{0}^{\infty }c*t\delta t$$

**Figure 2:**
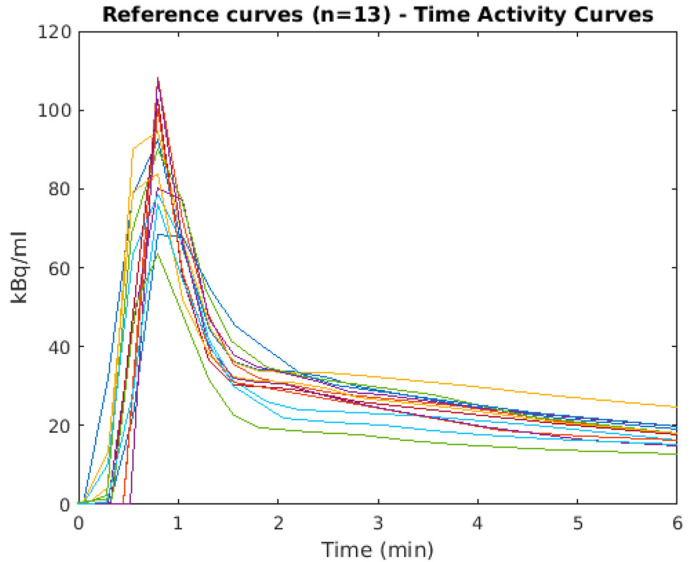
Peak area of the reference input time–activity curves (TACs) (11 arterial and 2 arterialized sampled plasma curves), selected according to step 2 of the model validation procedure, without the outlier after fitting the Feng equation (step 3a). The visualization of the curves in the figure is a linear interpolation, highlighting the raw data with the single time points. For clarity, each of the individual curves is plotted with a different color.

The fitted parameters were also analyzed, and one outlier was identified (having one fitted parameter > 2 SD than the reference set). Since the AUC % and MRT % absolute differences were on average ≤ 3% between the reference PA inputs and the fitted inputs, we proceeded the analysis with the selected reference curves (n = 13) (Figure 2). AUC and MRT are reported in Supplementary Tables S1–S3.

*(3b) Fit with Feng–Bayes model of reference PA inputs (only tail).*

In this step, we trained the model with the tail (5 min–100 min) of the reference input curves to estimate the initial peak (known for the reference curves) based on three defined constraints and the average fit parameters of the Feng input from *step 3*. 

*(4a) Finding constraints from linear regressions.* First, we correlated the Feng fit parameters and ratios between the parameters to other study characteristics (dose, anthropometric characteristics) and the curve parameters (e.g., max peak, AUCs). The best regression selected were: 

*(4a1) Linear regression 1:*

maxPA = 149.05 + 10943 ∗ (Ratio52), (4)

where maxPA is the maximum peak activity of the reference inputs and Ratio52 is the ratio between the parameter 5 and 2 of the fit with Feng input function (7 parameters in total, p5 corresponds to λ1 and p2 to A1 of the original publication nomenclature). The correlation is expected since both parameters are a characteristic of the first exponential that describes the inputs. The R^2^ of the linear model was 0.65.

*(4a2) Linear regression 2:*

p2 = 6357.91 − 520 ∗ (VSS), (5)

where p2 is the parameter 2 of the fit with Feng input function (which correspond to A1 of the original publication) and the volume of distribution at steady state (VSS) [50]:

VSS = (Dose ∗ MRT)/AUC,(6)

The R^2^ of this linear model was 0.46.

*(4a3) Linear regression 3:*

AUC(2–4 min) = 33.47 + 0.23 ∗ (Dose), (7)

where AUC (2–4 min) is the AUC between 2 and 4 min (keeping in mind that the ascending phase of the reference PA inputs start at about 2 min). The R^2^ of this linear model was 0.35.

The linear regression correlation coefficients and intercepts of Equations (4), (5) and (7) were fix into the model, working as constraints. 

*(4b) Feng–Bayes model specifications.*

The basic optimization function is “lsqnonlin” from Matlab’s optimization toolbox, executed with the trust region reflective algorithm.

The calculation of the objective function includes the subfunction Feng equation input with its seven parameters, to calculate the y data for every iteration (pseudo-code in Supplemental Material and Methods) and four conditions with fixed weights (regularization parameters) and two with fitted weights (p8, p9). The fitted parameters (p) were nine, seven from Feng input function and two regularization parameters. 

The first term is a Bayesian penalization based on noise, that is, the minimization of the difference between the fitted and original tail activity points normalized for the standard deviation of the sampled points of the population. The second term, like the first, minimizes the final fitted parameters instead.

The third, fourth and fifth terms are the constraints according to the linear regressions found in the steps 4a. The sixth term of the objective function minimizes the performance parameter MRT, between the fit and original tail.

*(4c) Visualization of fitted curves, computation of AUC and MRT percentual differences*

**Figure 3:**
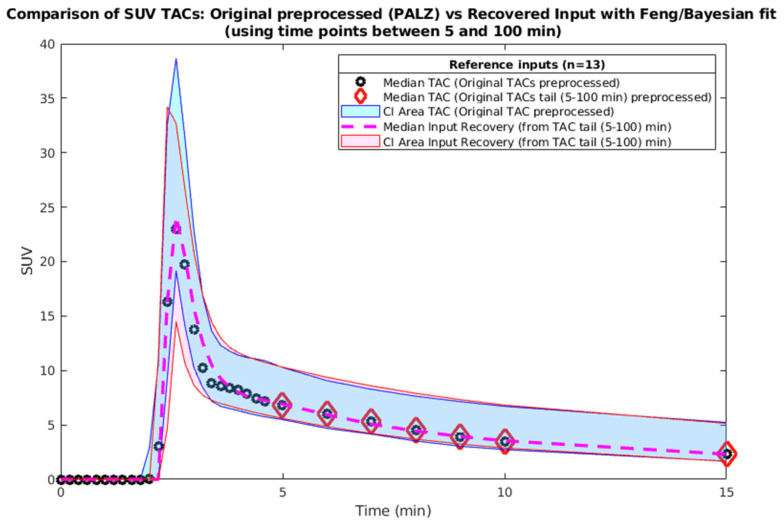
Reference input TACs—original and recovered by the Feng–Bayes model. The small circles are the data points of the median of the original TACs, the dashed line is the median of the recovered inputs and the areas are the confidence intervals (CI) 95% below and above median. Only the first portion of the inputs is visualized (0–15 min) in order to better visualize the overlap of the dashed line (the recovered inputs median) and the median of the original data points (circles). The data points marked with the diamonds (from 5 min) are the first portion of the input (5–100 min) fed into the IR Feng–Bayes model.

The resulting inputs were assessed visually to avoid un-realistic fits (Figure 3) and the performance statistics were computed (Supplementary Tables S1–S3). The goal was to keep the percentual difference of the performance parameters (maxSUV, AUC SUV and MRT SUV) between the recovered PA and original PA inputs below 5% on average. Achieving this, it required multiple iterations in loop through step 4d. 

*(4d) Adjustment of the weighting parameters and model comparisons*

The model weights of the different terms of the objective function were adjusted to minimize the maxSUV, AUC SUV and MRT SUV percentual differences. The resulting curves are visualized in Figure 3. The original and recovered input curves from the reference set had comparable AUC (d = 0.02, *p* > 0.05), maxSUV (d = 0.05, *p* > 0.05).

*(5) Developing a selection criterion of good- and poor-quality curves based on visualization.*

**Figure 4:**
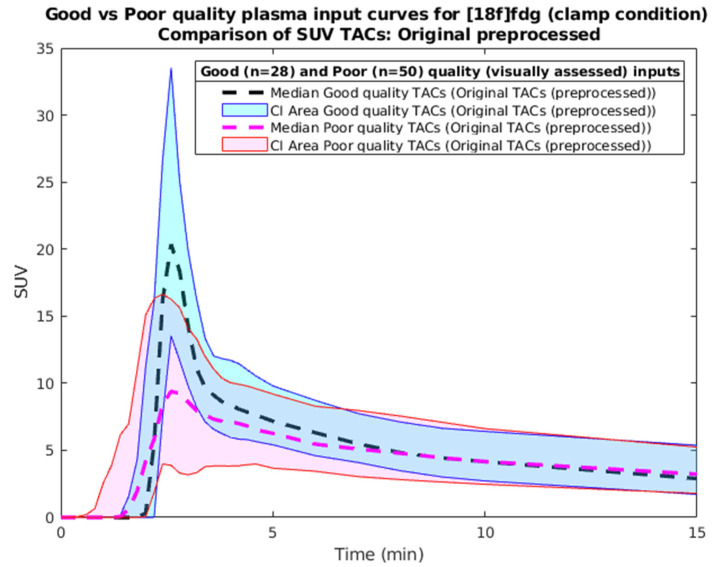
Early input TACs, good vs. poor quality by visual assessment.

The 78 training curves were visually assessed by the 1st author and categorized as of “acceptable” (N = 28) or “unacceptable” (N = 50) quality. The criteria were the height and shape (narrowness) of the peak compared to the population. Figure 4 shows the two populations. Then, a set of selection criteria was developed to resemble the visual categorization. Two variables resulted in the prediction of a good-quality curve: the maximum activity of the standard uptake value (SUV) curve, and the ratio between maximum activity value and the 5th min of the curve (peak/5th). The regression line used to select the inputs is the following:

Selection variable = −0.36 + 0.038 ∗ max SUV + 0.052 ∗ peak/5th,(8)

**Figure 5:**
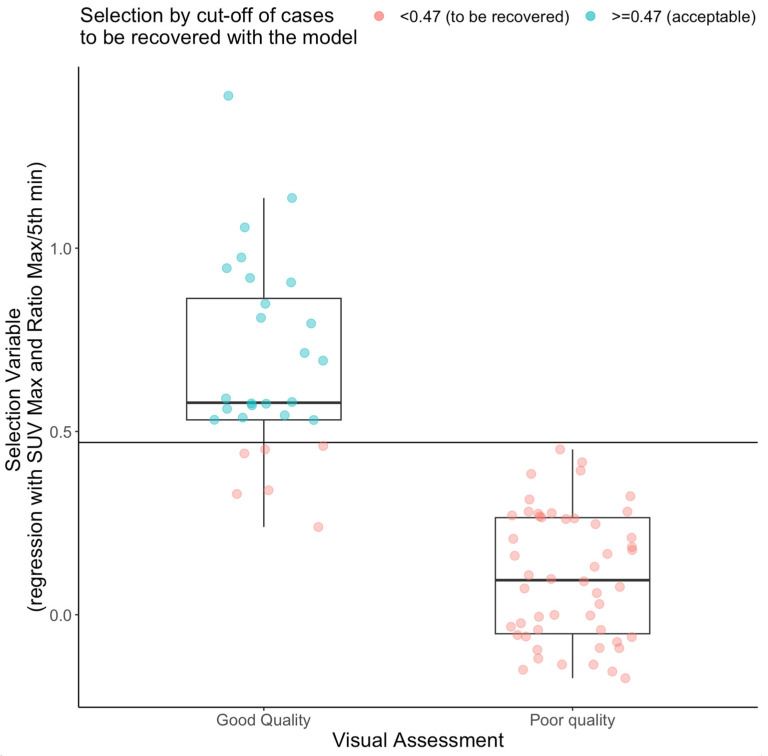
Selection criteria threshold validation. The regression value between the visual inspection and maximum SUV and maximum activity/5th min activity ratio was used to select poor-quality curves to recover with a cut-off of 0.47. Six curves that were originally considered to be of good quality by visual assessment were moved into the poor-quality pool to obtain the 56 TACs included in the training set of poor-quality curves.

A threshold of 0.47 resulted in the best separation between acceptable and unacceptable curves, with a misclassification of only 6 good curves (by visual assessment) as poor quality, as shown in Figure 5.

*(6) Fit with Feng–Bayes model of the training set with poor-quality PA inputs (only tails).*

**Figure 6:**
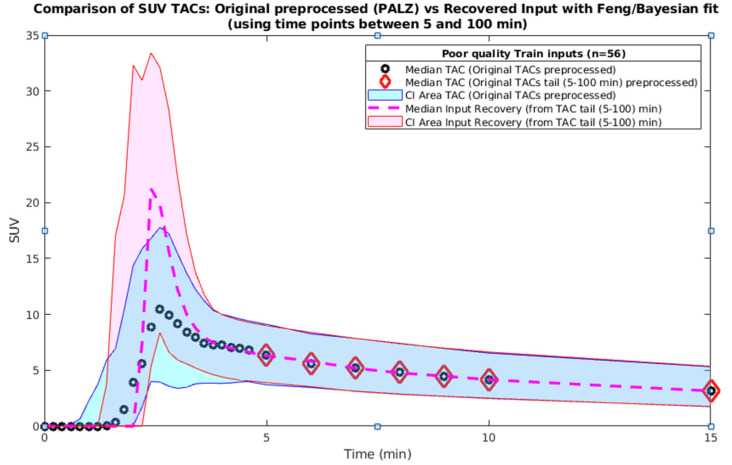
Poor-quality input TACs (training set, n = 56)—original and recovered by the Feng–Bayes model. The peak is recovered while the tail is kept similar to the original curves.

The 56 inputs that resulted in poor quality according to step 5 were recovered by fitting the model trained in step 4. The resulting inputs were visually assessed to avoid unrealistic fits and the model parameters were slightly adjusted accordingly. The resulting curves are visualized in Figure 6. The time correction to align the peak (step 1c) of all input curves was removed after the recovery procedure before the kinetic modeling. The performance statistics were computed (Supplementary Tables S1–S3) and noteworthy curves recovered from the training set were different from the original according to maxSUV (d = 3, *p* < 0.05) but not in AUC SUV (d = 0.12, *p* > 0.05).

*(7) Fit with (trained) Feng-Bayes function of the test set with poor-quality PA inputs (only tails).*

The 20 inputs that resulted in poor quality according to the selection criteria validated on step 5 from the 92 late inputs were recovered by fitting the model trained after step 6. The recovered input curves obtained were used for FUR modeling. 

### 2.5. Image Analysis

#### 2.5.1. Preprocessing

Each reconstructed early PET image was averaged across its frames and the resulting average image was used to acquire normalization parameters in conjunction with a brain [^18^F]FDG template in use at the Turku PET Centre. With these parameters, the PET dynamic images were then spatially normalized, and time–activity curves (TAC) were extracted from four regions of interest (ROI) (frontal, temporal, parietal and occipital lobes) obtained using MarsBaR (version 0.44, http://marsbar.sourceforge.net/, accessed on 1 January 2019).

#### 2.5.2. Compartmental Modeling—Model 3k

Input functions (original and IR) of the reference set and poor-quality training set were utilized with the extracted brain TACs to run a 3k compartmental model [51,52] with vascular fraction fixed to 0.05. The [^18^F]FDG PET tracer, once in the blood circulation, is considered in the compartment Cp (plasma), and the time–activity concentration in the plasma is also the input function that we aim to recover and is the amount of tracer available for each tissue to be taken up. The two-tissue compartmental model for [^18^F]FDG, which assumes that the tracer, once in the tissue (in this case the brain), can only be in two compartments, namely Cf (free (to diffuse back into the plasma or undergo phosphorylation)) and Cb (bound or metabolically trapped, due to the phosphorylation of fluorodeoxyglucose, which does not undergo further metabolization nor dephosphorylation for the time of the experiment). The rate of transfer of [^18^F]FDG between compartments in each direction is described by the so-called rate constants (1/min) and these are also used in the formulation of differential equations that analytically formulate the compartment model. The compartment Cp (activity in plasma) is connected to Cf in the tissue by K1 (inward in the tissue) and k2 (outward from the tissue), while k3 links Cf to Cb. The fitted parameters were K1, K1/k2 and k3, whilst ki was derived by K1 ∗ k3/(K1 + k2) [18]. Ki is the net influx constant rate of [^18^F]FDG in the tissue and the most important hyperparameter obtainable from this modeling for [^18^F]FDG in this context. The multiplication of Ki for the glucose concentration in the blood is used in PET studies (but not here) to obtain the glucose metabolic rate, the rate at which the cells of a specific tissue take up and utilize glucose.

#### 2.5.3. Maximal Theoretical K1 Threshold Calculation

In the reference set, we also had available PET studies with [^15^O]H_2_O, which were analyzed according to a previously published paper [5] to estimate the cerebral blood flow (CBF). With the following equations, the K1max was computed:

CPF = CBF × (1 − Htk),(9)

K1max = CPF ∗ Extraction (set to 1, upper limit),(10)

CPF, cerebral plasma flow, Htk, hematocrit

#### 2.5.4. Fractional Uptake Rate Modeling

Input functions (original and IR) of reference and poor-quality training and test sets were utilized with the extracted brain TACs to compute the FUR [16]. FUR is correlated to the Ki calculated with CM [18].

### 2.6. Statistical Analysis

Data are presented as mean ± SD (or confidence intervals (CI) 95%). A quantification of the effect size magnitude between the two groups of measurements is performed calculating the Cohen’s d and using the thresholds defined in Cohen (1992) [53]: |d| < 0.2 “negligible”, |d| < 0.5 “small”, |d| < 0.8 “medium” and otherwise “large”. Group comparisons of the results obtained between original and input recovery model curves were performed with paired *t*-test statistics corrected for multiple comparisons with Bonferroni method.

## 3. Results

### 3.1. Further Validation and Testing of the IR Model via Compartmental Modeling

**Figure 7:**
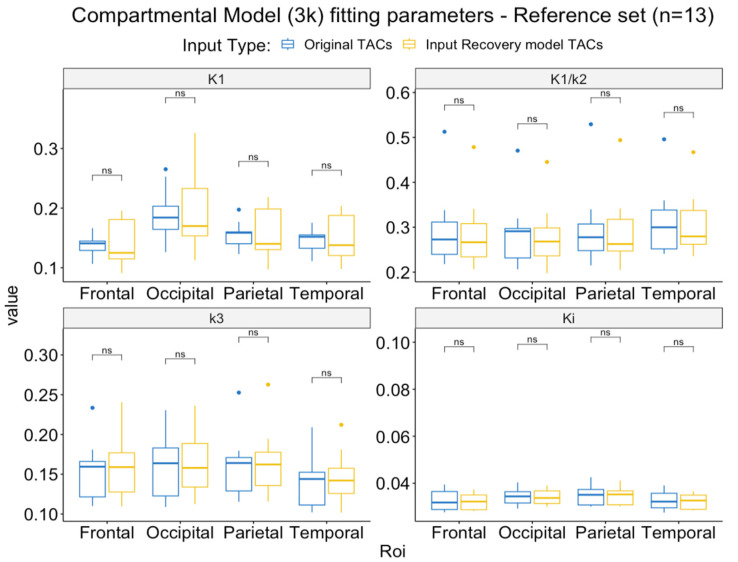
Comparison of the compartmental model parameters obtained with the original vs. recovered reference input functions. ns = *p* > 0.05.

The comparison of the compartmental model (3k) parameters obtained with original reference curves and IR model curves are shown Figure 7. The parameters were not different in any of the brain ROIs evaluated.

**Figure 8:**
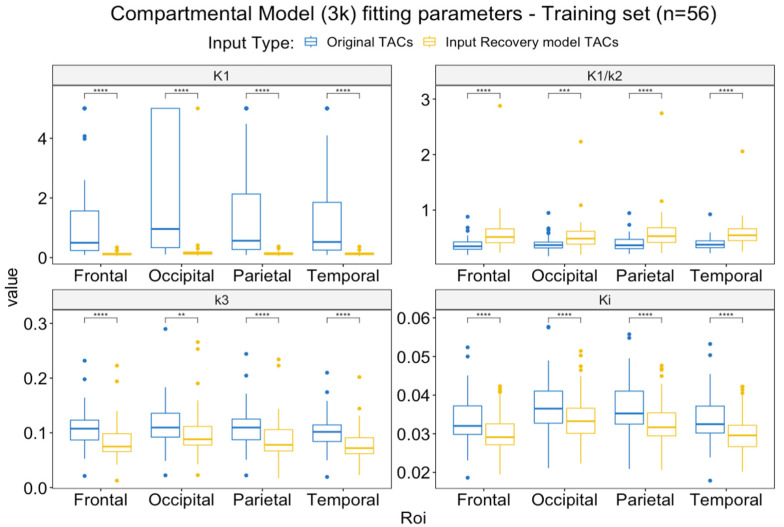
Comparison of the compartmental model parameters obtained with the original vs. recovered poor-quality input functions from the training set (n = 56). ** = *p* < 0.01, *** = *p* < 0.001, **** = *p* < 0.0001.

**Figure 9:**
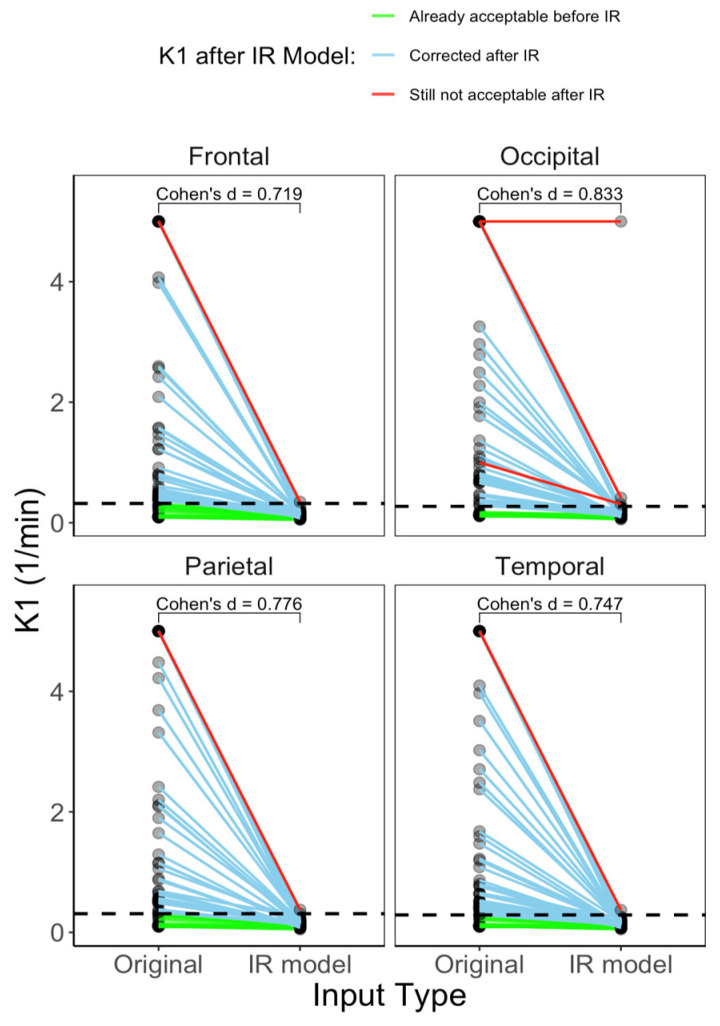
Compartmental model K1—IR modeling consistently reduces the values in the four ROIs analyzed bringing K1 values below the theoretical maximum K1 estimated from the perfusion studies of the reference curves. IR method failed to correct only one subject’s input according to the occipital ROI (no change in K1 estimate and saturating at the modeling boundary). In two other subjects, the recovered TACs produced a K1 that was closer to the threshold but above it.

Figure 8 shows the comparison between the compartmental model (3k) parameters obtained with the original (poor-quality) training curves and those obtained with the IR model curves. The parameters were different in all the brain ROIs evaluated. Additionally, the K1 parameter was confronted with the maximal theoretical K1 obtainable by the reference curves (based on their perfusion values) (Figure 9). Table 2 shows how K1 was originally out of the physiological range for 44/56 subjects in at least one ROI of the four considered, and after IR modeling, only three subjects remained with at least one ROI out of range, even though two of the three subjects’ K1 ended up being close to the threshold and the recovered input was still not close to the physiological range of K1 for only for one subject.

### 3.2. IR Model Further Validation and Testing via FUR

**Figure 10:**
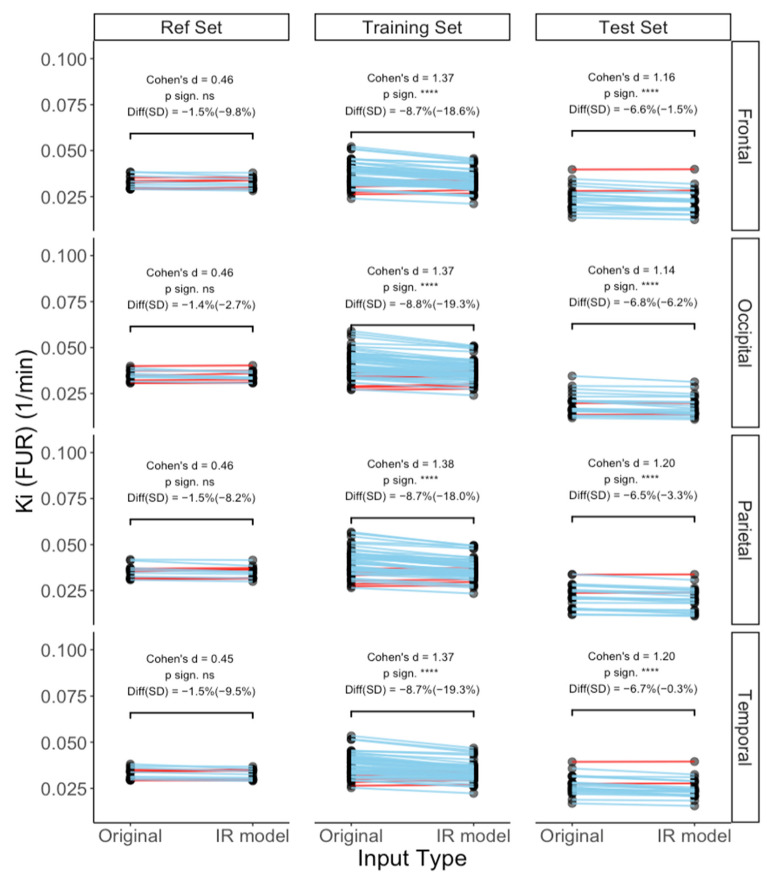
Fractional uptake rate—Ki parameter across datasets and in comparison, between original and recovered input functions—and the test set curves (n = 20 poor-quality curves) present a decrease in Ki similarly to that shown in the training set (n = 56). Red lines display a positive slope and the blue lines display a negative slope. ns = *p* > 0.05, **** = *p* < 0.0001.

Figure 10 shows the FUR results for reference, training, and test sets (only the poor-quality curves), respectively. The FUR results for the reference curves were not different between original and IR modeled curves, exhibiting small effect sizes (0.45–0.46 across ROIs) and minimal percentual differences (−1.4% to −1.5%). When comparing original and IR modeled of poor-quality input functions of the train set, the FUR differences had large effect sizes (1.37 to 1.38 across ROIs) and the differences between the original and IR model curves were −6.5 to −6.8% (across ROIs). The comparison of the test input curves also produced large effect sizes (1.14–1.2 across ROIs) and the differences between the FURs derived from original and IR model curves were between −8.7 and −8.8%. This suggests that employing the IR method for modeling poor-quality input functions leads to significant alterations in the input functions, subsequently exerting a notable impact on the quantification results.

## 4. Discussion

The main aim of the present study was to address one very common but inadequately addressed problem of PET analysis of poor-quality input function data. Good-quality input functions, especially when aiming at calculating compartmental model results, are essential, since this modeling is extremely sensitive to the shape of the initial part of the input function. In the proposed method, we set up a series of constraint terms, one of which is a Bayesian noise-penalizing one in an optimization routine that we called an “input recovery” (IR) method. 

### 4.1. Validation

We validated the IR model, first showing that the input functions selected as reference were successfully recovered solely based on the tail (5 min–100 min) of the input curve and the hereby-validated constraint parameters. Secondly, the CM parameters resulting from the reference input curves recovered via fitting were similar to the original reference curves. Thirdly, when applying the IR model to the early study curves (training set) that were selected for the poor quality group, we observed a recovery of the originally low peak of activity reflected by the significantly different maximal activity in SUV. The CM model parameters of the training set substantially improved in 44/56 subjects for which at least one ROI (out of the four brain ROIs analyzed) had a K1 that was above the theoretical maximum and the correction reduced the subjects that did not have physiological values to only 3/56 subjects, with 2/56 having a K1 right above the threshold. Fourthly, the IR model applied to the test set composed of late poor-quality curves also produced quantitative results (FUR) that were statistically different from the original poor-quality curves, testifying that the model did produce new corrected curves that yielded different results. 

### 4.2. Interpretation

We chose this method because the Bayesian penalization had been successfully applied in the field of PET image reconstruction [39] and we expected to succeed with its application to our problem. The results are in line with the expectations and confirmed that the chosen approach of input modeling can provide inputs with a significantly different peak (recovered) and physiologically plausible kinetic parameters derived from CM. This allows for the PET dynamic scans from time 0 from the injection (“early”) to carry the compartmental model analysis without the saturation of parameters (touching the boundaries) or errors produced by the solver not reaching convergence.

### 4.3. Comparison

This method was originally designed for correcting a pre-existing sampled input with the standard frequent sampling protocols but with a poor quality that resulted in the non-physiological peak shapes/heights or derived parameters via CM. Other groups have tried to reconstruct/fit the missing input functions with alternative methods to minimize the need for samples during the PET studies in association with a strong population input function equation [54,55,56,57,58,59,60]; these methods have a rather rigid input function shape scaled by the few samples withdrawn at the end of the study. We chose to place less emphasis on the population averages and instead developed a method that was more anchored in real data by incorporating a larger portion of the curve tail’s sampled datapoints. This approach also allows for a wider range of peak shapes through constraints on the relationships between the input function parameters.

A common example of methods to derive the input function is that from the images (IDIF) [34,36]. Compared to IDIF methods, our method is more expensive in terms of the data required, but extracting the input from the image—e.g., from the carotid arteries—requires that: (a) the region has to be in the field of view; (b) the resolution has to be adequate; (c) the region to extract should not be affected by spill-over effects from neighboring regions; and (d) the tracer should have a good noise/signal ratio (which is not true, for example, for [^18^H]H_2_O).

### 4.4. Applicability

So, this method becomes helpful when IDIF methods are not applicable. Moreover, it promotes the reuse of data acquired with old scanners, aligning with sustainable research principles that advocate to not always strive to acquire more data but also reuse old data and explore with these using new hypotheses. On the top of this, the IR method ensures the fitting of high-quality curves while minimizing errors generated during quantification by poor-quality curves.

### 4.5. Further Developments

Future developments for this approach involve: (1) refining the sampling protocol to reduce the number of samples required while still achieving optimal results—it is important to note that this aspect was not initially included in the scope of the planned analyses; (2) a direct comparison of the current method with more widely accepted image-derived input functions (IDIFs), which was unfeasible due to the limitations in the available image data. Most of the images were late scans, lacking the initial peak of tracer activity, and the early scans were acquired using outdated PET scanners with insufficient spatial resolution to extract a carotid input function. 

### 4.6. Limitations

One limitation inherent to the study’s design is the unavailability of the true input curve for the poor-quality sampled inputs. As discussed earlier, deriving the input from image was not an option; therefore, the internal validation for this study was the physiological plausibility of the K1 obtained via CM based on the reference studies for which the perfusion studies were available. Also, it remains to be determined whether patients’ characteristics—rather than the tail characteristics per se—may influence the input function kinetics. Since there are no studies investigating the sex differences in the [^18^F]FDG tracer kinetics in the brain during clamp (and this study did not aim to investigate this question), we cannot exclude that such differences exist; given that our population being predominately female, the results might not be generalizable. It must be noted that our statistical analyses are conducted within subjects, which mitigate such limitations. An additional limitation could be the manual visual pre-selection of the input as good or poor quality, as first step to create the selection criteria.

## 5. Conclusions

In conclusion, the validation steps of the proposed input recovery (IR) modeling approach against a reference set of curves proved to be useful and successful. The recovered curves were similar to the original ones in the reference set. In the training set (poor-quality curves), the peak region and the kinetic parameters from the compartmental model (CM) were different from the original curves, and the peak was “recovered” (higher) and, for example, K1 became physiologically plausible in almost all cases (except 3/56). As PET imaging is expensive and involves exposing the study subjects and volunteers to ionizing radiation, all these efforts are jeopardized in cases of poor-quality input curves. Therefore, we suggest that the method described herein could be employed to provide meaningful [^18^F]FDG-PET results. 

## Figures and Tables

**Table 1:** General table by set.

	Reference Set (N = 13)	Training Set (N = 65)	Test Set (N = 92)	Total (N = 170)
Sex				
Female	11 (84.6%)	54 (83.1%)	61 (66.3%)	126 (74.1%)
Male	2 (15.4%)	11 (16.9%)	31 (33.7%)	44 (25.9%)
Age				
Mean (SD)	48.4 (12.0)	46.2 (9.4)	61.2 (13.2)	54.5 (13.8)
Range	31.6–66.0	23.2–62.0	20.5–79.8	20.5–79.8
BMI				
Mean (SD)	25.7 (5.2)	33.2 (8.0)	27.7 (4.5)	29.7 (6.7)
Range	20.1–39.9	20.3–50.9	19.0–41.0	19.0–50.9
Dose				
Mean (SD)	250.8 (33.2)	188.7 (17.2)	176.9 (22.1)	187.1 (28.7)
Range	187.0–289.0	147.0–278.0	133.0–237.0	133.0–289.0
Original input (type)				
Arterial	11 (84.6%)	2 (3.1%)	0 (0.0%)	13 (7.6%)
Arterialized	2 (15.4%)	63 (96.9%)	7 (7.6%)	72 (42.4%)
Peak from image—aortic arch	0 (0.0%)	0 (0.0%)	24 (26.1%)	24 (14.1%)
Peak from image—left ventricle	0 (0.0%)	0 (0.0%)	61 (66.3%)	61 (35.9%)
Input quality (good/poor)				
Poor quality	0 (0.0%)	56 (86.2%)	20 (21.7%)	76 (44.7%)
Good quality	13 (100.0%)	9 (13.8%)	72 (78.3%)	94 (55.3%)
Scan type (early/late)				
Early	13 (100.0%)	65 (100.0%)	0 (0.0%)	78 (45.9%)
Late	0 (0.0%)	0 (0.0%)	92 (100.0%)	92 (54.1%)
Dataset from:				
Bucci M et al., JCM 2023 [3]	0 (0.0%)	0 (0.0%)	39 (42.4%)	39 (22.9%)
Hirvonen J et al., Diabetes 2011 [8]	0 (0.0%)	16 (24.6%)	0 (0.0%)	16 (9.4%)
Honkala SM et al., JCBFM 2018 [7]	0 (0.0%)	0 (0.0%)	13 (14.1%)	13 (7.6%)
Latva-Rasku A et al., Diabetes 2018 [6]	0 (0.0%)	0 (0.0%)	22 (23.9%)	22 (12.9%)
Lindroos MM et al., Brain 2009 [5]	11 (84.6%)	2 (3.1%)	0 (0.0%)	13 (7.6%)
Tuulari JJ et al., Diabetes 2013 [4]	2 (15.4%)	47 (72.3%)	0 (0.0%)	49 (28.8%)
Brain data unpublished courtesy of Viljanen AP	0 (0.0%)	0 (0.0%)	7 (7.6%)	7 (4.1%)
Brain data unpublished courtesy of Virtanen KA	0 (0.0%)	0 (0.0%)	11 (12.0%)	11 (6.5%)

**Table 2:** Number of input curves resulting K1 above maximal theoretical threshold in the training set (n = 56).

Roi	Original	Input Recovery Model
Frontal	37	1
Occipital	44	3
Parietal	40	1
Temporal	40	1

## Data Availability

Code is available to be shared upon request to the corresponding author. Per local legislation and patient consent, redistribution of the data is not permitted.

## Supplementary Materials

The following supporting information can be downloaded at: https://www.mdpi.com/article/10.3390/metabo14020114/s1, Additional description of Methods (Pseudocodes of the main functions), Table S1: General Table by Dataset; Table S2: General Table by Input Quality; Table S3: General Table by Input/Peak origin.

## Abbreviations

AUC, area under the curve; AUMC, area under the first moment curve; BGU, brain glucose uptake; BMI, body mass index; CI, confidence interval; CM, compartmental modeling; FDG, [18F]-fluorodeoxyglucose; FDR, false discovery rate; FUR, fractional uptake rate; IR, input recovery; MRT, mean retention time; NS, not significant; PA, peak–aligned; PET, positron emission tomography; SD: standard deviation; SUV, standardized uptake value; TAC, time–activity curve; UAV, upper adjacent value.
