# Supplementary material for: Kinetic Modeling of Brain [18-F]FDG Positron Emission Tomography Time Activity Curves with Input Function Recovery (IR) Method
Source: Metabolites. 2024 Feb 8;14(2):114. doi: 10.3390/metabo14020114 (PMC10890269; doi:10.3390/metabo14020114)
Supplement: Supplementary file 1 [file metabolites-14-00114-s001.zip › Supplemental Material and Methods.pdf]

SUPPLEMENTAL MATERIALS AND METHODS

At the core of the input recovery method lies the non-linear optimization (lsqnonlin, MATLAB) of the objective function (Supp. Eq.2), which has as input

1) a vector *p* with 9 parameters (7 parameters of the Feng sub-function (Supp. Eq.1), and p(8) and p(9) weights for the constraint %5 and %6)
2) fit_data (the structure with all fixed and updated parameters, such as tail samples and performance parameters, explained in detail below)

While the optimization is ongoing, inside the objective function, the y data (fitted recovered input) is calculated for every iteration until convergence.

Supp. Eq. 1 (Feng Input):

(p(2)*(t - p(1))-p(3)-p(4))*exp(p(5)*(t - p(1))) + p(3)*exp(p(6)*(t - p(1))) + p(4)*exp(p(7)*(t - p(1)))

Supp Eq. 2 (pseudo code of the objective function to be minimized at each iteration):

```
Ojective_function=
[fit_data.weight1.*((fit_data.latesamples_y-y(p,fit_data,t))./fit_data.latesamples_standarddev.); %1
fit_data.weight2.*((fit_data.parameters_average-p(1:7))./fit_data.parameters_standarddev);     %2
fit_data.weight3.*(((m1_intercept+m1_r.*(p(5)./p(2)))-max_peak));                               %3
fit_data.weight4.*(((m2_intercept+m3_r.*vss)-p(2))));                                            %4
p(8).*(((m3_intercept+m3_r.*dose)-AUC_2_to_4_min));                                              %5
p(9).*(MRT_fitted_curve_5_to_100_min - MRT_original_curve_5_to_100_min)];                        %6
```

y() is the function that produces the recovered fitted curve with parameters p (Feng function), the fit_data (structure) and t the time column. In fit_data the used fields by the objective function are: weights1-4 that have been optimized and fixed during training.

latesamples_y: the list of the activity concentration of the late samples

latesamples_standarddev: the standard deviation of the activity concentration of the late samples

parameters_average: the average of the parameters of Feng input before the fitting (fixed by training)

parameters_std: the standard deviation of the parameters of Feng input before the fitting (fixed by training)

m1_intercept and m1_r: refer to the regression of Step 4a1 in the manuscript

m2_intercept and m2_r: refer to the regression of Step 4a2 in the manuscript

m3_intercept and m3_r: refer to the regression of Step 4a3 in the manuscript

max_peak: maximal activity at the peak

vss: volume of distribution at steady state (see Eq.5 in the main text)

dose: injected dose in MBq

AUC_2_to_4_min = is recalculated at each iteration

MRT_fitted_curve_5_to_100_min = mean retention time of the tail is recalculated at each iteration
