# Supplementary material for: Kinetic Modeling of Brain [18-F]FDG Positron Emission Tomography Time Activity Curves with Input Function Recovery (IR) Method
Source: Metabolites. 2024 Feb 8;14(2):114. doi: 10.3390/metabo14020114 (PMC10890269; doi:10.3390/metabo14020114)
Supplement: Supplementary file 1 [file metabolites-14-00114-s001.zip › Supp_Tables.pdf]

Table S1. General Table by Dataset

	Reference set (N=13)	Train set (N=56)	Test set (N=20)	Total (N=89)	p value
**Scan type (Early/Late)**					< 0.001
Early	13 (100.0%)	56 (100.0%)	0 (0.0%)	69 (77.5%)	
Late	0 (0.0%)	0 (0.0%)	20 (100.0%)	20 (22.5%)	
**Original_Input_Type**					< 0.001
Arterial	11 (84.6%)	0 (0.0%)	0 (0.0%)	11 (12.4%)	
Arterialized	2 (15.4%)	56 (100.0%)	7 (35.0%)	65 (73.0%)	
Peak from Image - aortic arch	0 (0.0%)	0 (0.0%)	12 (60.0%)	12 (13.5%)	
Peak from Image - LV	0 (0.0%)	0 (0.0%)	1 (5.0%)	1 (1.1%)	
**Input quality (Good/Poor)**					< 0.001
Poor quality	0 (0.0%)	56 (100.0%)	20 (100.0%)	76 (85.4%)	
Good quality	13 (100.0%)	0 (0.0%)	0 (0.0%)	13 (14.6%)	
**Gender**					0.540
f	11 (84.6%)	48 (85.7%)	15 (75.0%)	74 (83.1%)	
m	2 (15.4%)	8 (14.3%)	5 (25.0%)	15 (16.9%)	
**Age**					0.615
Mean (SD)	48.4 (12.0)	46.3 (9.5)	44.7 (11.7)	46.2 (10.3)	
Range	31.6 - 66.0	23.2 - 62.0	20.5 - 69.0	20.5 - 69.0	
**BMI**					< 0.001
Mean (SD)	25.7 (5.2)	33.8 (7.9)	26.9 (6.0)	31.1 (8.0)	
Range	20.1 - 39.9	20.3 - 50.9	19.6 - 41.0	19.6 - 50.9	
**Dose**					< 0.001
Mean (SD)	250.8 (33.2)	186.5 (10.4)	191.7 (25.8)	197.1 (29.4)	
Range	187.0 - 289.0	147.0 - 214.0	149.0 - 237.0	147.0 - 289.0	
**Max SUV (Original)**					< 0.001
Mean (SD)	25.0 (6.1)	10.6 (4.5)	11.8 (3.0)	13.0 (6.7)	
Range	19.1 - 40.0	3.5 - 18.4	7.7 - 17.7	3.5 - 40.0	
**Max SUV (IR (recovered))**					0.190
Mean (SD)	24.7 (4.6)	26.3 (5.4)	23.9 (4.8)	25.5 (5.2)	
Range	18.8 - 35.2	15.4 - 39.9	17.6 - 33.4	15.4 - 39.9	
**Max SUV (Original-IR perc diff)**					< 0.001
Mean (SD)	0.9 (16.5)	193.5 (128.9)	111.4 (48.8)	146.9 (125.5)	
Range	-19.9 - 36.8	44.6 - 549.0	14.1 - 198.1	-19.9 - 549.0	
**AUC SUV (Original)**					0.025
Mean (SD)	146.2 (69.2)	176.1 (70.4)	130.8 (50.1)	161.6 (68.4)	
Range	89.6 - 316.4	75.6 - 338.4	74.0 - 253.4	74.0 - 338.4	
**AUC SUV (IR (recovered))**					0.018
Mean (SD)	147.8 (67.6)	184.8 (71.6)	139.2 (50.7)	169.2 (69.3)	
Range	90.0 - 313.4	82.2 - 346.3	83.2 - 254.1	82.2 - 346.3	
**AUC SUV (Original-IR perc diff)**					0.003
Mean (SD)	1.6 (2.6)	5.6 (4.1)	7.2 (6.4)	5.3 (4.8)	
Range	-2.5 - 6.8	-2.6 - 13.2	-0.8 - 24.9	-2.6 - 24.9	
**MRT SUV (Original)**					0.001
Mean (SD)	20.3 (4.6)	26.0 (5.3)	22.9 (5.3)	24.5 (5.6)	

	Reference set (N=13)	Train set (N=56)	Test set (N=20)	Total (N=89)	p value
Range	15.2 - 29.2	15.6 - 42.2	15.0 - 33.6	15.0 - 42.2	
**MRT SUV (IR (recovered))**					0.003
Mean (SD)	19.8 (5.0)	24.5 (5.4)	20.7 (5.3)	22.9 (5.6)	
Range	14.7 - 29.2	15.0 - 37.7	13.2 - 31.5	13.2 - 37.7	
**MRT SUV (Original-IR perc diff)**					0.003
Mean (SD)	-3.1 (3.1)	-6.1 (5.2)	-9.7 (6.9)	-6.5 (5.7)	
Range	-9.0 - 1.5	-24.0 - 1.2	-25.3 - 0.5	-25.3 - 1.5	

Table S2. General Table by Input Quality

	Poor quality (N=76)	Good quality (N=13)	Total (N=89)	p value
**Set type**				< 0.001
Reference set	0 (0.0%)	13 (100.0%)	13 (14.6%)	
Train set	56 (73.7%)	0 (0.0%)	56 (62.9%)	
Test set	20 (26.3%)	0 (0.0%)	20 (22.5%)	
**Scan type (Early/Late)**				0.036
Early	56 (73.7%)	13 (100.0%)	69 (77.5%)	
Late	20 (26.3%)	0 (0.0%)	20 (22.5%)	
**Original_Input_Type**				< 0.001
Arterial	0 (0.0%)	11 (84.6%)	11 (12.4%)	
Arterialized	63 (82.9%)	2 (15.4%)	65 (73.0%)	
Peak from Image - aortic arch	12 (15.8%)	0 (0.0%)	12 (13.5%)	
Peak from Image - LV	1 (1.3%)	0 (0.0%)	1 (1.1%)	
**Gender**				0.878
f	63 (82.9%)	11 (84.6%)	74 (83.1%)	
m	13 (17.1%)	2 (15.4%)	15 (16.9%)	
**Age**				0.420
Mean (SD)	45.8 (10.0)	48.4 (12.0)	46.2 (10.3)	
Range	20.5 - 69.0	31.6 - 66.0	20.5 - 69.0	
**BMI**				0.008
Mean (SD)	32.0 (8.0)	25.7 (5.2)	31.1 (8.0)	
Range	19.6 - 50.9	20.1 - 39.9	19.6 - 50.9	
**Dose**				< 0.001
Mean (SD)	187.9 (15.9)	250.8 (33.2)	197.1 (29.4)	
Range	147.0 - 237.0	187.0 - 289.0	147.0 - 289.0	
**Max SUV (Original)**				< 0.001
Mean (SD)	10.9 (4.1)	25.0 (6.1)	13.0 (6.7)	
Range	3.5 - 18.4	19.1 - 40.0	3.5 - 40.0	
**Max SUV (IR (recovered))**				0.557
Mean (SD)	25.6 (5.4)	24.7 (4.6)	25.5 (5.2)	
Range	15.4 - 39.9	18.8 - 35.2	15.4 - 39.9	
**Max SUV (Original-IR perc diff)**				< 0.001
Mean (SD)	171.9 (118.8)	0.9 (16.5)	146.9 (125.5)	
Range	14.1 - 549.0	-19.9 - 36.8	-19.9 - 549.0	
**AUC SUV (Original)**				0.385
Mean (SD)	164.2 (68.3)	146.2 (69.2)	161.6 (68.4)	
Range	74.0 - 338.4	89.6 - 316.4	74.0 - 338.4	
**AUC SUV (IR (recovered))**				0.232
Mean (SD)	172.8 (69.4)	147.8 (67.6)	169.2 (69.3)	
Range	82.2 - 346.3	90.0 - 313.4	82.2 - 346.3	
**AUC SUV (Original-IR perc diff)**				0.002
Mean (SD)	6.0 (4.8)	1.6 (2.6)	5.3 (4.8)	
Range	-2.6 - 24.9	-2.5 - 6.8	-2.6 - 24.9	
**MRT SUV (Original)**				0.003

	Poor quality (N=76)	Good quality (N=13)	Total (N=89)	p value
Mean (SD)	25.2 (5.5)	20.3 (4.6)	24.5 (5.6)	
Range	15.0 - 42.2	15.2 - 29.2	15.0 - 42.2	
**MRT SUV (IR (recovered))**				0.029
Mean (SD)	23.5 (5.6)	19.8 (5.0)	22.9 (5.6)	
Range	13.2 - 37.7	14.7 - 29.2	13.2 - 37.7	
**MRT SUV (Original-IR perc diff)**				0.019
Mean (SD)	-7.1 (5.8)	-3.1 (3.1)	-6.5 (5.7)	
Range	-25.3 - 1.2	-9.0 - 1.5	-25.3 - 1.5	

Table S3. General Table by Input/Peak Origin

	Arterial (N=11)	Arterialized (N=65)	Peak from Image - aortic arch (N=12)	Peak from Image - LV (N=1)	Total (N=89)	p value
**Set type**						< 0.001
Reference set	11 (100.0%)	2 (3.1%)	0 (0.0%)	0 (0.0%)	13 (14.6%)	
Train set	0 (0.0%)	56 (86.2%)	0 (0.0%)	0 (0.0%)	56 (62.9%)	
Test set	0 (0.0%)	7 (10.8%)	12 (100.0%)	1 (100.0%)	20 (22.5%)	
**Scan type (Early/Late)**						< 0.001
Early	11 (100.0%)	58 (89.2%)	0 (0.0%)	0 (0.0%)	69 (77.5%)	
Late	0 (0.0%)	7 (10.8%)	12 (100.0%)	1 (100.0%)	20 (22.5%)	
**Input quality (Good/Poor)**						< 0.001
Poor quality	0 (0.0%)	63 (96.9%)	12 (100.0%)	1 (100.0%)	76 (85.4%)	
Good quality	11 (100.0%)	2 (3.1%)	0 (0.0%)	0 (0.0%)	13 (14.6%)	
**Gender**						0.041
f	9 (81.8%)	57 (87.7%)	8 (66.7%)	0 (0.0%)	74 (83.1%)	
m	2 (18.2%)	8 (12.3%)	4 (33.3%)	1 (100.0%)	15 (16.9%)	
**Age**						0.012
Mean (SD)	48.5 (11.9)	46.7 (9.3)	39.6 (11.0)	69.0 (NA)	46.2 (10.3)	
Range	31.6 - 66.0	23.2 - 62.0	20.5 - 54.8	69.0 - 69.0	20.5 - 69.0	
**BMI**						< 0.001
Mean (SD)	24.1 (2.9)	33.7 (7.6)	23.8 (3.5)	23.6 (NA)	31.1 (8.0)	
Range	20.1 - 29.0	20.3 - 50.9	19.6 - 32.1	23.6 - 23.6	19.6 - 50.9	
**Dose**						< 0.001
Mean (SD)	261.8 (21.3)	190.4 (15.0)	177.9 (11.7)	149.0 (NA)	197.1 (29.4)	
Range	232.0 - 289.0	147.0 - 237.0	159.0 - 200.0	149.0 - 149.0	147.0 - 289.0	
**Max SUV (Original)**						< 0.001
Mean (SD)	23.3 (4.2)	11.3 (6.0)	12.9 (3.2)	10.9 (NA)	13.0 (6.7)	
Range	19.1 - 31.0	3.5 - 40.0	8.1 - 17.7	10.9 - 10.9	3.5 - 40.0	
**Max SUV (IR (recovered))**						0.496
Mean (SD)	23.9 (3.7)	26.0 (5.3)	24.4 (5.9)	23.1 (NA)	25.5 (5.2)	
Range	18.8 - 28.6	15.4 - 39.9	17.6 - 33.4	23.1 - 23.1	15.4 - 39.9	
**Max SUV (Original-IR perc diff)**						< 0.001
Mean (SD)	4.0 (16.0)	181.1 (126.3)	95.5 (50.3)	110.8 (NA)	146.9 (125.5)	
Range	-16.5 - 36.8	-19.9 - 549.0	14.1 - 198.1	110.8 - 110.8	-19.9 - 549.0	
**AUC SUV (Original)**						0.010
Mean (SD)	126.2 (46.5)	175.9 (70.2)	123.1 (46.4)	84.6 (NA)	161.6 (68.4)	
Range	89.6 - 252.1	75.6 - 338.4	74.0 - 220.7	84.6 - 84.6	74.0 - 338.4	
**AUC SUV (IR (recovered))**						0.009

	Arterial (N=11)	Arterialized (N=65)	Peak from Image - aortic arch (N=12)	Peak from Image - LV (N=1)	Total (N=89)	p value
Mean (SD)	128.5 (46.0)	183.9 (70.8)	132.7 (49.8)	95.8 (NA)	169.2 (69.3)	
Range	90.0 - 251.3	82.2 - 346.3	83.2 - 236.9	95.8 - 95.8	82.2 - 346.3	
**AUC SUV (Original-IR perc diff)**						0.004
Mean (SD)	2.0 (2.6)	5.2 (4.1)	8.3 (7.4)	13.2 (NA)	5.3 (4.8)	
Range	-2.5 - 6.8	-2.6 - 13.2	-0.8 - 24.9	13.2 - 13.2	-2.6 - 24.9	
**MRT SUV (Original)**						< 0.001
Mean (SD)	19.1 (3.8)	25.9 (5.1)	21.7 (6.0)	24.8 (NA)	24.5 (5.6)	
Range	15.2 - 29.2	15.6 - 42.2	15.0 - 33.6	24.8 - 24.8	15.0 - 42.2	
**MRT SUV (IR (recovered))**						< 0.001
Mean (SD)	18.4 (3.9)	24.4 (5.2)	19.4 (5.7)	18.5 (NA)	22.9 (5.6)	
Range	14.7 - 29.0	15.0 - 37.7	13.2 - 31.5	18.5 - 18.5	13.2 - 37.7	
**MRT SUV (Original-IR perc diff)**						< 0.001
Mean (SD)	-3.9 (2.7)	-5.9 (5.1)	-10.4 (6.7)	-25.3 (NA)	-6.5 (5.7)	
Range	-9.0 - 0.2	-24.0 - 1.5	-23.5 - 0.5	-25.3 - -25.3	-25.3 - 1.5	
